# Hospital Cookery

**Published:** 1924-10

**Authors:** A La Lanterne


### CORRESPONDENCE.

### Hospital Cookery.

*To the Editor of* The Hospital and Health Review.

Sir,—The number of angels who have come down to earth and "taken to" cooking is, to the knowledge of all of us, very small; but it might have been supposed that this select band would have descended upon the hospitals instead of going to kings' houses or illustrating the tables of millionaires, which would appear to be their favoured destination. Can anything be imagined more stodgy and unappetising than the fare ordinarily placed before hospital patients? Very often it is not even hot, and almost invariably it is banal and unimaginative to the last degree. One does not expect the Café Riche in hospital, and I know quite well the difficulty of cooking for large numbers of people; even in one's own club one avoids, if one is wise, the "cut from the joint." But assuredly variety in food should assist convalescence, and it is the business of a hospital to get its patients cured and sent home as quickly as possible.

What is to be done about it? Cookery, the first and most sublime of all the arts, is daily murdered by hired assassins who, with cynical effrontery, call themselves cooks. Yet vengeance tarries!

A La Lanterne.

